# 

**Published:** 1896-01

**Authors:** 


					﻿ESTABLISHED 1855.
8UB8CRIPTI0N, $2.00.
ATLANTA
JWEDIGflli AflD SURGIGflli
JOURNAL.	j
VOLUME XII.
NEW SERIES.
Atlanta, Ga., January,	No. 11\
				

